# Supplementary material for: Meta-Analysis and Gene Set Enrichment Relative to ER Status Reveal Elevated Activity of MYC and E2F in the “Basal” Breast Cancer Subgroup
Source: PLoS One. 2009 Mar 9;4(3):e4710. doi: 10.1371/journal.pone.0004710 (PMC2650420; doi:10.1371/journal.pone.0004710)
Supplement: Dataset S1 — GSEA results from first screen. The file contains a zipped archive of the HTML results of the second GSEA screen. Reports for enriched or depleted datasets may be accessed from the “index.html” link within the my_analysis.GseaPreranked.1219975950625.rpt.zip ZIP archive. Enrichment plots are available for each of the 50 most enriched and depleted gene sets. (13.32 MB ZIP) [file pone.0004710.s001.zip › my_analysis.GseaPreranked.1219975950625.rpt.zip!//HDACI_COLON_TSA2HRS_UP.html]

Details for gene set HDACI\_COLON\_TSA2HRS\_UP[GSEA]

|  || Dataset | zSco.ERneg.to.ERpos\_collapsed |
| Phenotype | NoPhenotypeAvailable |
| Upregulated in class | na\_neg |
| GeneSet | HDACI\_COLON\_TSA2HRS\_UP |
| Enrichment Score (ES) | -0.5053814 |
| Normalized Enrichment Score (NES) | -2.026927 |
| Nominal p-value | 0.0 |
| FDR q-value | 0.007824799 |
| FWER p-Value | 0.148 |
Table: GSEA Results Summary

  

Fig 1: Enrichment plot: HDACI\_COLON\_TSA2HRS\_UP      
 Profile of the Running ES Score & Positions of GeneSet Members on the Rank Ordered List

  

| PROBE | GENE SYMBOL | GENE\_TITLE | RANK IN GENE LIST | RANK METRIC SCORE | RUNNING ES | CORE ENRICHMENT || 1 | SAT1 |  |  | 514 | 4.322 | 0.0012 | No |
| 2 | ETV5 |  |  | 1073 | 3.387 | -0.0096 | No |
| 3 | MT1H |  |  | 2140 | 2.422 | -0.0676 | No |
| 4 | PLAG1 |  |  | 2201 | 2.387 | -0.0500 | No |
| 5 | KCNA1 |  |  | 2559 | 2.169 | -0.0569 | No |
| 6 | GNL1 |  |  | 3040 | 1.913 | -0.0754 | No |
| 7 | MBNL2 |  |  | 3321 | 1.785 | -0.0800 | No |
| 8 | CDKN1C |  |  | 3739 | 1.609 | -0.0966 | No |
| 9 | CYB5B |  |  | 3763 | 1.599 | -0.0836 | No |
| 10 | VEGF |  |  | 3911 | 1.549 | -0.0803 | No |
| 11 | MAP1LC3B |  |  | 4713 | 1.249 | -0.1292 | No |
| 12 | NEUROD6 |  |  | 5581 | 0.949 | -0.1857 | No |
| 13 | BIRC4 |  |  | 6051 | 0.794 | -0.2137 | No |
| 14 | EPB41 |  |  | 6428 | 0.672 | -0.2358 | No |
| 15 | JARID1C |  |  | 6662 | 0.602 | -0.2478 | No |
| 16 | ROCK1 |  |  | 6704 | 0.585 | -0.2455 | No |
| 17 | PC |  |  | 6791 | 0.556 | -0.2469 | No |
| 18 | ISG15 |  |  | 6857 | 0.533 | -0.2468 | No |
| 19 | INPP5E |  |  | 7393 | 0.347 | -0.2840 | No |
| 20 | ZUBR1 |  |  | 7541 | 0.291 | -0.2923 | No |
| 21 | GLRX |  |  | 8308 | -0.001 | -0.3500 | No |
| 22 | SERPINE1 |  |  | 8947 | -0.283 | -0.3955 | No |
| 23 | ID2 |  |  | 9049 | -0.335 | -0.4000 | No |
| 24 | P4HB |  |  | 9771 | -0.707 | -0.4478 | No |
| 25 | PER1 |  |  | 10374 | -1.077 | -0.4832 | No |
| 26 | HSPA1A |  |  | 10471 | -1.135 | -0.4800 | No |
| 27 | C5 |  |  | 10561 | -1.215 | -0.4755 | No |
| 28 | CDH1 |  |  | 10717 | -1.328 | -0.4749 | No |
| 29 | ZC3H12A |  |  | 10964 | -1.542 | -0.4792 | No |
| 30 | SLC20A1 |  |  | 11029 | -1.593 | -0.4693 | No |
| 31 | GNAI2 |  |  | 11509 | -2.046 | -0.4865 | Yes |
| 32 | LOC137886 |  |  | 11704 | -2.225 | -0.4806 | Yes |
| 33 | NEU1 |  |  | 11789 | -2.317 | -0.4655 | Yes |
| 34 | TSNAX |  |  | 12040 | -2.628 | -0.4601 | Yes |
| 35 | NECAP1 |  |  | 12150 | -2.774 | -0.4427 | Yes |
| 36 | FOSB |  |  | 12385 | -3.084 | -0.4318 | Yes |
| 37 | FOS |  |  | 12389 | -3.085 | -0.4036 | Yes |
| 38 | CDH11 |  |  | 12438 | -3.164 | -0.3780 | Yes |
| 39 | SDC4 |  |  | 12541 | -3.322 | -0.3550 | Yes |
| 40 | TRIM52 |  |  | 12737 | -3.709 | -0.3354 | Yes |
| 41 | FLNB |  |  | 12764 | -3.781 | -0.3025 | Yes |
| 42 | CALCOCO1 |  |  | 12786 | -3.834 | -0.2687 | Yes |
| 43 | PIP5K2C |  |  | 12796 | -3.883 | -0.2335 | Yes |
| 44 | SPARCL1 |  |  | 12889 | -4.108 | -0.2025 | Yes |
| 45 | DAAM1 |  |  | 12945 | -4.313 | -0.1668 | Yes |
| 46 | RHOB |  |  | 13091 | -4.795 | -0.1335 | Yes |
| 47 | TNCRNA |  |  | 13139 | -4.984 | -0.0910 | Yes |
| 48 | GLI3 |  |  | 13213 | -5.527 | -0.0455 | Yes |
| 49 | RHBDF1 |  |  | 13243 | -5.792 | 0.0058 | Yes |
Table: GSEA details [plain text format]

  

Fig 2: HDACI\_COLON\_TSA2HRS\_UP: Random ES distribution      
 Gene set null distribution of ES for **HDACI\_COLON\_TSA2HRS\_UP**
